# Supplementary material for: Su(H) Modulates Enhancer Transcriptional Bursting in Prelude to Gastrulation
Source: Cells. 2024 Oct 24;13(21):1759. doi: 10.3390/cells13211759 (PMC11545809; doi:10.3390/cells13211759)
Supplement: Supplementary file 1 [file cells-13-01759-s001.zip › cells-3237810-supplementary/cells-3237810-supplementary.pdf]

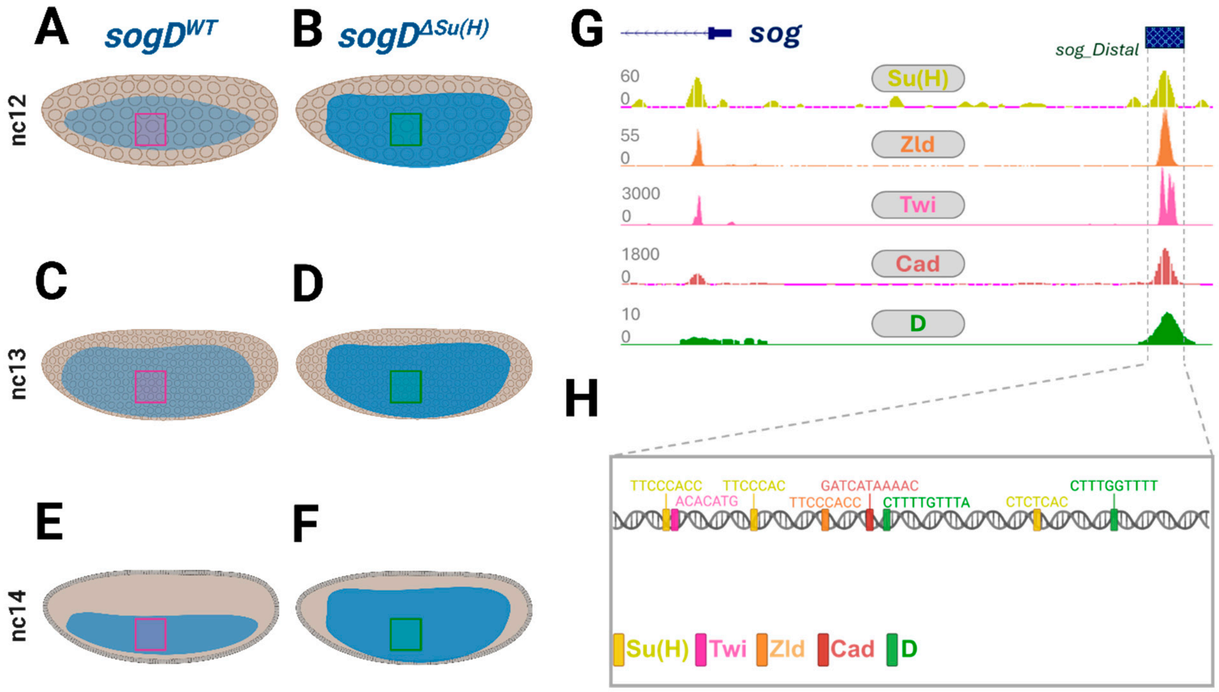


**Supplemental Figure S1.** *sogD* dynamics, illustrated. (**A**–**F**) Illustrations of sogD-driven reporter expression. (**G**) ChIP-seq binding data for, Suppressor of hairless (Su(H)), Zelda (Zld), Twist (Twi), Caudal (Cad), and Dichaete (**D**) showing occupancy of these factors at the *sogD* locus. (**H**) Binding motif map of these factors along the sogD enhancer locus. sogDSu(H) enhancers contain mutations in each of the Su(H) sites to prevent Su(H) binding. See Videos S1–S2.

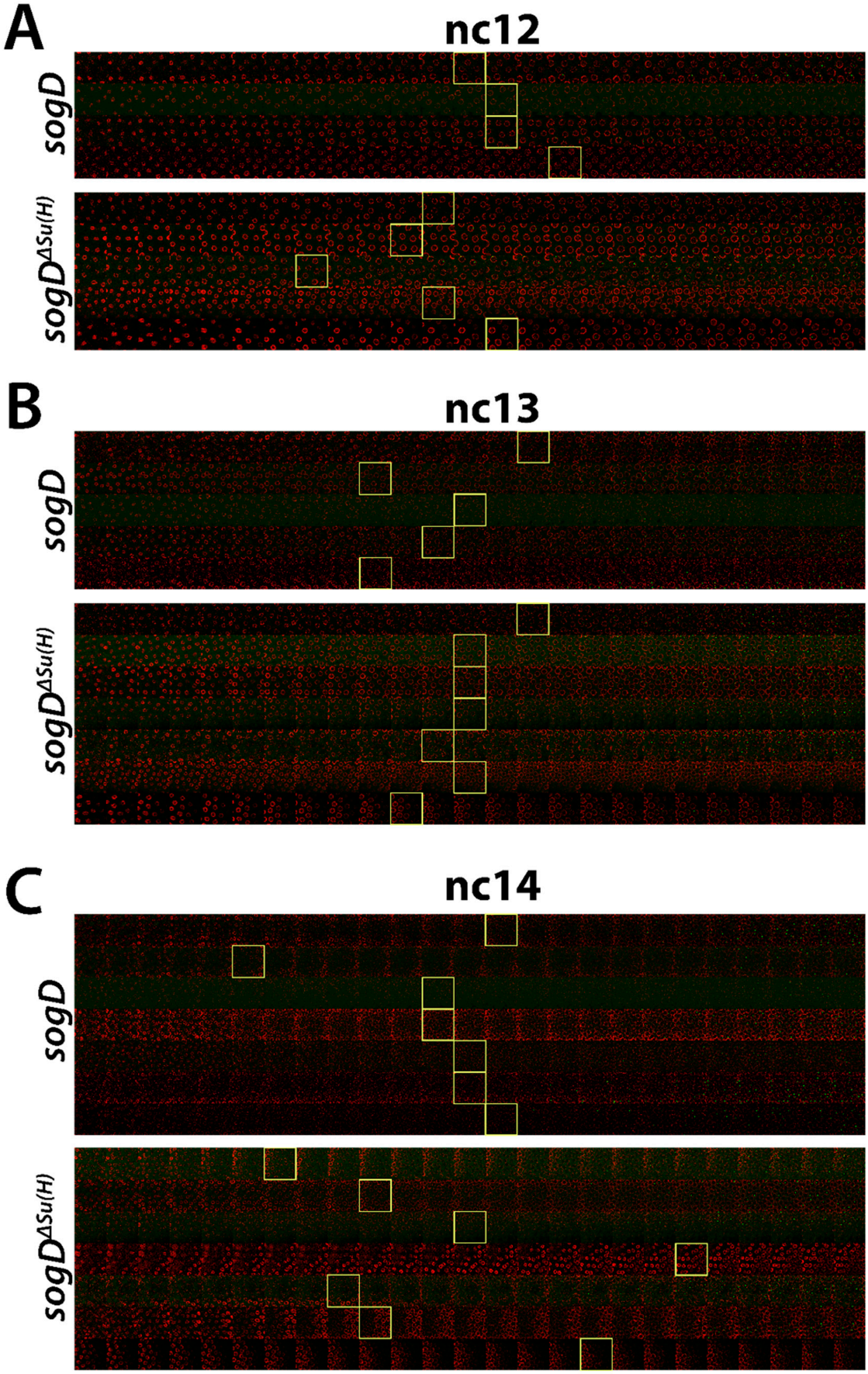


**Supplemental Figure S2.** First 25 frames from each movie. (**A**) Top: The first 25 frames of nc12 of the 4 wt *sogD*:MS2 movies. Bottom: The first 25 frames of nc12 of the 5 *sogD* $^{\Delta Su(H)}$:MS2 movies. (**B**) Top: The first 25 frames of nc13 of the 6 wt *sogD:MS2* movies. Bottom: The first 25 frames of nc13 of the 7 *sogD* $^{\Delta Su(H)}$:MS2 movies. **(C)** Top: The first 25 frames of nc14 of the 7 wt *sogD*:MS2 movies.

Bottom: The first 25 frames of nc14 of the 7 sogD ΔSu(H):MS2 movies. Yellow boxes denote the frame of first dot.

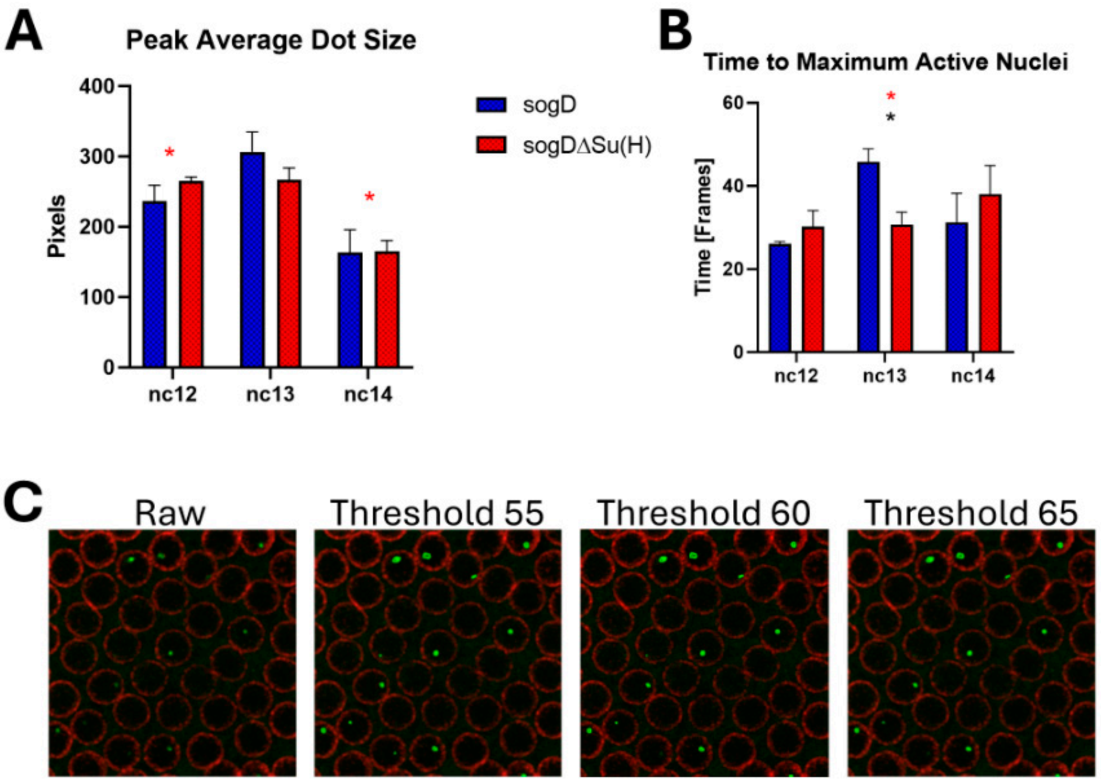


**Supplemental Figure S3.** (**A**,**B**) Su(H) occupancy at *sogD* increases variance in maximum average dot size at nc12 (*: $p = 0.0302$) and at nc14 (*: $p = 0.0311$) and in time to reach maximum active nuclei (*: $p = 0.0216$). (C) Raw vs masked dot images comparing intensity thresholds. .

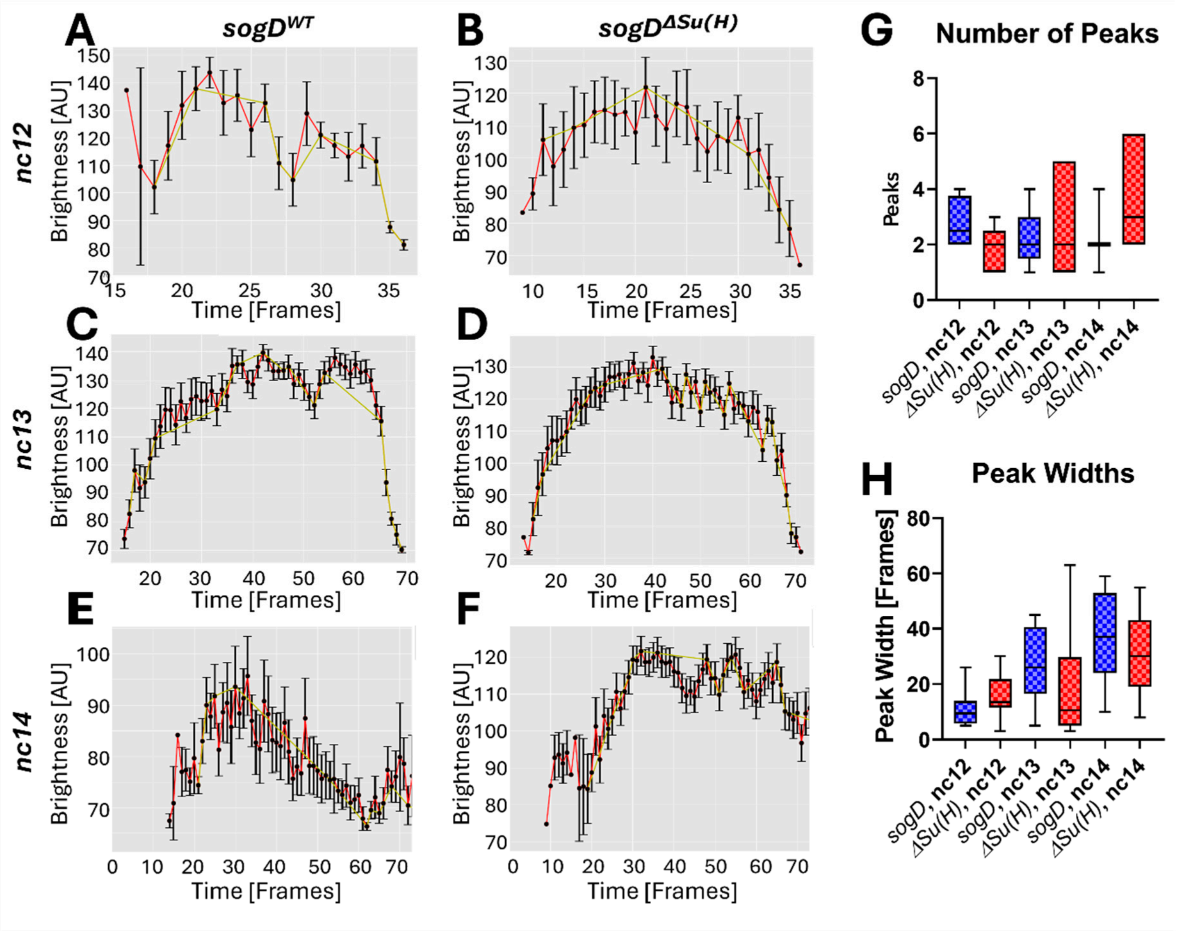


**Supplemental Figure S4.** Su(H) regulation of tissue-level transcriptional fluctuations. (**A**,**B**) Plots of transcriptional fluctuations in representative mutant and wt reporter embryos at nc12. (**C**,**D**) Plots of transcriptional fluctuations in representative mutant and wt reporter embryos at nc13. (**E**,**F**) Plots of transcriptional fluctuations in representative mutant and wt reporter embryos at nc14. (**G**) Boxplots of transcriptional peak frequency (total number of peaks per movie per nc). (**H**) Boxplots of transcriptional fluctuation peaks duration (width between troughs per movie per nc). **A**–**F** line plots represent combined frame-wide brightness averages of all fluorescent dots at that timepoint (frame) across all embryos. Yellow lines in **A**–**F** map identified peaks (transcriptional fluctuation, determined by transitions between non-overlapping SEM ranges).
